# Encouraging Students and Trainees to Write

**DOI:** 10.31662/jmaj.2022-0134

**Published:** 2022-11-30

**Authors:** Soichiro Saeki

**Affiliations:** 1 Center Hospital of the National Center for Global Health and Medicine, Tokyo, Japan

**Keywords:** Medical education, Medical English, Undergraduate, Postgraduate, Japan

## Abstract

Publications from Japan have been on the decline, and this tendency is expected to continue as the country’s population decreases. During the novel coronavirus disease (COVID-19) pandemic, it was discovered that Japanese medical trainees published much fewer papers than trainees from other nations. This issue must be addressed by the entire Japanese medical community.

Trainees have the potential to contribute to the medical community through their publishing process presenting fresh perspectives and communicating accurate information to the public through the use of social media. Furthermore, trainees themselves would benefit as they would become more enhanced by deeply and critically considering the contents and the trends of publications worldwide, which would promote further implementation of evidence-based medicine.

Therefore, medical educators and students should be motivated and encouraged to write by offering enough instructional and publication opportunities. Such measures would be to (1) train students in “medical writing” as part of the medical curriculum; (2) encourage medical students and trainees to submit of manuscripts, especially in the section of the letters, opinions, and case reports; (3) guarantee trainees time and resources to write; (4) provide as constructive reviews and comments as possible as an educational opportunity for trainees; and (5) motivate trainees to write.

Such hands-on training would necessitate significant efforts of the trainees, instructors, and publishers. However, if we cannot invest in fostering future resources now, we may not be able to hope for increase in the amount of research published from Japan. The future lies in everybody’s hands.

Many medical researchers are still haunted by the old adage, “Publish or perish.” For a long time, the value of publishing new manuscripts has been underlined. However, it has been noticed that Japanese research is on the decline ^(1)^. Although it may appear inevitable, given that the number of researchers in Japan is expected to fall as the population of Japan declines, some countermeasures should be taken.

To tackle this issue, we propose to promote the writing short manuscripts such as letters or opinion articles at an early stage in the career, such as during medical school or residencies. During the COVID-19 pandemic, it was observed that publications by medical students in Japan are less compared to those in other countries ^(2)^. We believe that young trainees in Japan also have the capacity to produce articles that will benefit the medical and scientific communities, and by encouraging them to write in a welcoming environment, we can boost the number of present and future publications from Japan.

Trainees can make a significant contribution to the scientific community for three reasons. Firstly, young scholars would be able to offer fresh viewpoints to the table. In *Nature Medicine*, medical students acknowledged inequalities they confront during their career-building process ^(3)^, igniting a new debate on how under- and postgraduate education should be conducted. The entire medical community would benefit if trainees were encouraged to write, as they would be able to contribute objective thoughts from unique viewpoints. Secondly, young trainees may be able to communicate critical medical, public health messages to the general population. The importance of social media as an important source of information has been emphasized during the COVID-19 pandemic, and the young generation has been recognized as an important member of the medical society for disseminating accurate information in a meaningful manner ^(4)^. Encouraging young trainees to publish increases the visibility and permeability of medical information they write, read, and share, which would be a benefit to the publisher and the public, as well as the medical society. Thirdly, increased Japanese publications would contribute to the formation of a medical consensus that comprehensively incorporates the perspectives of the Japanese medical society. Although clinical settings may differ between Japan and the rest of the world, if publications from trainees increase from Japan, new insights into medical events and studies from Japan can be distributed to the world accordingly, which may encourage researchers from other nations to include viewpoints from the Japanese medical community. This may aid studies or perspective articles in bringing the setting or viewpoints closer to the environment of Japan, potentially increasing the generalizability of such manuscripts to Japanese settings.

So, what can we do to encourage young Japanese trainees to write? The author has five suggestions.

Firstly, students should be trained in “medical writing” as part of the medical curriculum. Medical English is taught in medical schools in Japan, with a sufficient number of classes required. On the other hand, the current curriculum primarily focuses on learning medical terminology and conducting consultations in English. Although such education is important, particularly because patients from various backgrounds are visiting Japanese medical facilities, and have limited Japanese proficiency, some of such hours should be included to provide students with the opportunity to learn how to write medical papers. This should include a lecture on the overview of how medical manuscripts are structured and the key rules in writing, such as what information should not be left out from medical manuscripts. If possible, hands-on training on writing in paragraphs, focused on the structures with topic and supporting sentences, could be offered. As the entrance examinations for medical schools in Japan typically require a high level of English, students who are interested should be able to educate themselves once they receive a proper guidance to get started.

Secondly, educators and editors of journals published in Japan should encourage medical students and trainees to submit of manuscripts, especially in the section of the letters, opinions, and case reports. Such actions would promote university instructors’ educational process of to teach their students how to write, as publications should also contribute to promoting the careers of the teachers as well.

Thirdly, trainees should be guaranteed time and resources to write. It has been noted that one of the reasons for the decrease in the number of articles from Japan is due to the lack of young physicians in university hospitals not being able to commit to research as before ^(5)^, and such opportunities should be secured, even if it may be for a short amount of time. The action of providing such an opportunity would encourage trainees to address their clinical duties while on the lookout for potential research opportunities, which should foster the mindset that could last throughout their careers, to create new research questions. Furthermore, trainees themselves would benefit as they would become more enhanced by deeply and critically considering the contents and the trends of publications worldwide, which would promote further implementation of evidence-based medicine.

Fourthly, publishers, editors, and reviewers should provide as constructive reviews and comments as possible, so that the submission process serves as an educational opportunity for trainees, regardless of whether the manuscript itself was accepted or rejected. Furthermore, writing a letter in response to a published article will provide trainees with an educational opportunity to foster critical reading ^(6)^, which would also promote their abilities.

Finally, trainees should be motivated to write. This can be promoted by hosting writing workshops ^(7)^ or with lectures from clinical experts on how writing has benefited them. Having one colleague succeed in publishing may also encourage their fellow colleagues to try for themselves; thus, promoting the initial step would be the key.

To summarize, encouraging Japanese medical trainees to write would benefit both the trainee and the instructor, as well as the entire Japanese medical community. Such experiences should help trainees to publish full papers in the future and thus increase the future amount of research published from Japan. However, this would necessitate time and effort on the part of both the trainees and the Japanese medical society. The future of Japanese medical research lies in everybody’s hands.

## Article Information

### Conflicts of Interest

None

### Acknowledgement

The author thanks the educational opportunities provided by his instructors and fellow colleagues throughout his training.

### Author Contributions

The author is responsible for the conceptualization and the writing of the manuscript.

### Approval by Institutional Review Board (IRB)

Not applicable
